# Supplementary material for: The Systems Biology Research Tool: evolvable open-source software
Source: BMC Syst Biol. 2008 Jun 29;2:55. doi: 10.1186/1752-0509-2-55 (PMC2446383; doi:10.1186/1752-0509-2-55)
Supplement: Additional file 1 — SBRT Archive. An archive of the current version of the Systems Biology Research Tool. [file 1752-0509-2-55-S1.zip › sbrt-1.4.0/doc/developers_guide/api/sbrt/kernel/math/stat/comp/class-use/KendallTau.html]

Uses of Class sbrt.kernel.math.stat.comp.KendallTau


|  |  |  |
| --- | --- | --- |
| **Overview** \| **Package** \| **Class** \| **Use** \| **Tree** \| **Deprecated** \| **Index** \| **Help** | |  |
| PREV   NEXT | **FRAMES**    **NO FRAMES**     **All Classes** |  |


---


## **Uses of Class sbrt.kernel.math.stat.comp.KendallTau**

| Packages that use KendallTau | |
| --- | --- |
| **sbrt.kernel.math.stat.comp** | Provides classes and interfaces for performing statistical computations. |
| **sbrt.shell.mng.stat** | Provides classes and interfaces for creating statistical process managers. |

Uses of KendallTau in sbrt.kernel.math.stat.comp

| Subclasses of KendallTau in sbrt.kernel.math.stat.comp | |
| --- | --- |
| `class` | `KendallTauA<V>`             This class is used to compute Kendall's Tau_A_ correlation statistic. |
| `class` | `KendallTauB<V>`             This class is used to compute Kendall's Tau_B_ correlation statistic. |

Uses of KendallTau in sbrt.shell.mng.stat

| Methods in sbrt.shell.mng.stat that return KendallTau | |
| --- | --- |
| `static KendallTau<java.lang.String>` | `KendallTauManager.parseCorrelator(java.lang.String stat)`             Returns the Kendall's Tau statistic computer corresponding to the provided string value. |

---


|  |  |  |
| --- | --- | --- |
| **Overview** \| **Package** \| **Class** \| **Use** \| **Tree** \| **Deprecated** \| **Index** \| **Help** | |  |
| PREV   NEXT | **FRAMES**    **NO FRAMES**     **All Classes** |  |


---
